# Comparing taxes as a percentage of sugar-sweetened beverage prices in Latin America and the Caribbean

**DOI:** 10.1016/j.lana.2022.100257

**Published:** 2022-04-21

**Authors:** Maxime Roche, Miriam Alvarado, Rosa Carolina Sandoval, Fabio da Silva Gomes, Guillermo Paraje

**Affiliations:** a Centre for Health Economics and Policy Innovation (CHEPI), Imperial College Business School, Exhibition Rd, London SW7 2AZ, UK; b MRC Epidemiology Unit, Cambridge Biomedical Campus, University of Cambridge School of Clinical Medicine, Institute of Metabolic Science, Cambridge, UK; c Pan American Health Organization (PAHO), Washington D.C., USA; d Universidad Adolfo Ibáñez, Santiago, Chile

**Keywords:** Noncommunicable diseases, Nutrition policy, Sugar-sweetened beverages, Obesity, Fiscal policies, Health economics, CIF, Cost, insurance, and freight, IMF, International Monetary Fund, LAC, Latin America and the Caribbean, NCD, Noncommunicable disease, PAHO, Pan American Health Organization, PPP, Purchasing power parity, SSB, Sugar-sweetened beverage, VAT, Value added or sales taxes, WHO, World Health Organization

## Abstract

**Background:**

Excise taxes can be used to reduce the consumption of sugar-sweetened beverages (SSBs), an important preventable risk factor for noncommunicable diseases. This study aimed to compare novel standardized indicators of the level of taxes applied on SSBs as a percentage of the price across beverage categories in Latin America and the Caribbean.

**Methods:**

We used a method developed by the Pan American Health Organization and adapted from the World Health Organization's tobacco tax share. The analysis focused on the most sold brand of five categories of non-alcoholic beverages. Data were collected by surveying ministries of finance and reviewing tax legislation in effect as of March 2019.

**Findings:**

Of the 27 countries analyzed, 17 applied excise taxes on SSBs. Of these, median excise taxes represented the highest share of the price for large sugar-sweetened carbonated drinks (6·5%) and the lowest for energy drinks (2·3%). In countries where excise taxes were applied on bottled waters, tax incidence exceeded the one applied on most SSBs. Overall, excise tax shares were higher in Latin America than in the Caribbean. Including all other indirect taxes (e.g., value added tax), median total tax shares were between 12·8% and 17·5%. At least two countries earmarked part of SSB excise tax revenues for health purposes.

**Interpretation:**

Excise tax levels are generally low in the region. From a public health perspective, tax rates could be increased, and tax designs improved (e.g., excluding bottled waters). The method describe here provides a feasible and informative way to monitor SSB taxation and could be replicated in other regions and over time.

**Funding:**

Bloomberg Philanthropies through the Global Health Advocacy Incubator.

**Research in context**

**Evidence before this study**

The World Health Organization (WHO) recommends reducing sugar consumption through effective taxation of sugar-sweetened beverages (SSBs). Latin America and the Caribbean (LAC) has one of the highest levels of SSB consumption in the world. Twenty one out of the 33 LAC Member States of the Pan American Organization (PAHO) apply national level excise taxes on SSBs. A previous qualitative analysis highlighted high heterogeneity and suboptimal tax designs from a health perspective. Nevertheless, no standardized metric has been used to measure the level of taxes applied on SSBs across countries. On the other hand, tobacco taxes have been monitored biennially by WHO across all Member States since 2008 using a tax share indicator, allowing to compare tax levels across countries and monitor trends.

**Added value of this study**

This study presents the first region-wide estimation of a standardized and comparable metric of tax levels applied on non-alcoholic beverages. Using PAHO's methodology derived from WHO's method for the monitoring of tobacco taxes, we report low tax levels on the five SSB categories selected in LAC, with median excise taxes (when applied) and total taxes, respectively representing 2·3-6·5% and 12·8-17·5% of the final retail price. Excise tax shares were higher for sugar-sweetened carbonated drinks than other SSBs and in some countries, were relatively higher for bottled waters than for most SSBs. At least two countries earmarked part of SSB excise tax revenues for health purposes.

**Implications of all evidence available**

Our findings show low tax levels and confirm a significant level of policy incoherence in tax designs from a public health point of view. There are opportunities for reforms to increase tax rates and improve tax designs to increase the impact of existing excise taxes on SSB consumption. Tax share estimates provide a powerful tool to compare taxation levels across countries and beverage categories, assess the impact of a variety of tax designs, and inform policy making. This approach could be applied more widely and replicated over time.

Alt-text: Unlabelled box

## Introduction

Latin America and the Caribbean (LAC) has some of the highest consumption levels of sugar-sweetened beverages (SSBs) in the world. In the Caribbean and Central America, average daily consumption among adults is more than three times the global average.^1^ SSB consumption has been associated with the development of a number of non-communicable diseases (NCDs), including type 2 diabetes, coronary heart disease and hypertension.^2^, ^3^, ^4^, ^5^ Their consumption represents an important driver of the obesity epidemic,^6^^,^^7^ which is pervasive in LAC.^8^ In 2010, SSBs were estimated to account for 184,000 global deaths annually.^9^ Affordability of SSBs has increased in the majority of countries between 1990 and 2016, and this trend is particularly strong in low- and middle-income countries.^10^^,^^11^ The attributable burden of disease associated with SSBs, the projected increases in NCD-related costs, and the ability to effectively and selectively target SSBs with health policies have contributed to growing global interest in curbing SSB consumption.

Excise taxes represent one major policy tool that can be used to target SSB consumption.^12^ An excise tax is a tax on a selected good, generally collected from the manufacturer, wholesaler, or importer. Such taxes allow policy makers to target and raise the price of specific products, making them relatively less affordable than other goods and services. Excise taxes can be used to correct market failures (externalities and internalities), when the price of unhealthy products – such as SSBs – does not reflect the full social and individual costs associated with their consumption.^13^ There is growing evidence of the effectiveness of SSB excise taxes in reducing SSB consumption,^14^ particularly in LAC.^15^, ^16^, ^17^, ^18^

As of 2019, 73 countries worldwide applied excise taxes on SSBs.^19^ In LAC, such taxes are applied in 21 countries (out of the 33 LAC Member States of the World Health Organization). However, these taxes differ widely in terms of structure (e.g., type, uniformed vs. tiered), rate, 'base’, or the product's value on which the tax rate applies (e.g., the producer price), or the taxable unit in the case of taxes defined as a monetary amount per volume or sugar content, and products on which they are applied. Many are not optimized to achieve health goals.^20^ Given heterogeneity in tax design, how can we monitor and compare tax levels applied on SSBs across countries and time as well as between beverage categories? It is not straightforward to compare, for example, Mexico's 1 peso per liter tax with the 10% tax applied in Barbados, nor to tease apart tax effects across beverage categories of varied sugar content and volume sizes. It is of interest to develop and monitor standardized and comparable estimates of the level of indirect taxes applied on SSBs, particularly excise taxes. Such monitoring could enable improved comparisons between various tax designs and their impacts on prices.^21^

Since 2008, the World Health Organization (WHO) has estimated a tobacco tax share indicator for all WHO Member States biennially. This indicator, defined as the share of indirect taxes in the retail price of a 20-cigarette pack of the most sold brand, informs whether the retail price of cigarettes is comprised mostly by production costs and the manufacturer's or distributor's profits, or by indirect taxes.^22^ A similar indicator has also been employed in the literature to monitor alcohol tax levels.^23^^,^^24^ Tobacco tax share estimates have been used to monitor regional trends, guide decisions about tax design, and track industry pricing strategies in response to tax changes.^25^, ^26^, ^27^ While tax share estimates do not tell the full story about tobacco taxation, they are crucial for efforts to monitor the use of this policy over time and across countries. A similar metric is needed to measure SSB tax levels.^21^ However, estimating such a metric for a different and more heterogeneous group of products requires careful consideration and entails various trade-offs.

The aims of this analysis are (1) to estimate a standardized and comparable tax share indicator to provide the first comprehensive region-wide assessment of SSB tax levels in LAC and (2) to compare the level of taxes applied on SSBs across countries, by beverage categories (including between SSBs and non-SSBs) and tax designs. The paper also investigates the earmarking of revenue from excise taxes on SSBs. It discusses the policy implications of the findings and the need for the development of a systematized and periodic global monitoring of tax levels applied on SSBs.

## Methods

This analysis is based on the method developed by the Pan American Health Organization (PAHO) and reviewed by peer-researchers as well as officials from LAC ministries of finance in 2018.^28^^,^^29^ It is an adaptation of the well-established method used by WHO to estimate the tobacco tax share indicator.^22^ Below, we present a brief summary of the study design and methods. A more in-depth description is available elsewhere.^29^

### The indicator

The total tax share indicator results from dividing the total amount of indirect taxes by the final retail price faced by the consumer (inclusive of all indirect taxes, as applicable). This is equivalent to summing the share of each type of indirect taxes in the final retail price, as shown in Eq. (1) below:

(1) $$S_{t}=\frac{A_{vat}+A_{as}+A_{av}+A_{id}+A_{o}}{P}=S_{vat}+S_{as}+S_{av}+S_{id}+S_{o}$$

Where $S_{t}$ represents the total share of taxes in the final retail price (or total tax share indicator). $S_{vat}$, $S_{as}$, $S_{av}$, $S_{id}$, and $S_{o}$ represent, respectively the share of value added or sales taxes (VAT), amount-specific excise taxes, *ad valorem* excise taxes, import duties, and other indirect taxes in the final retail price – which is defined for each tax type as the amount of the tax over the final retail price. $A_{vat}$, $A_{as}$, $A_{av}$, $A_{id}$, and $A_{o}$ represent, respectively the amount of VAT, amount-specific excise taxes, *ad valorem* excise taxes, import duties, and other indirect taxes. *P* represents the final retail price faced by the consumer (inclusive of all indirect taxes, as applicable).

Calculating $A_{vat}$ and $A_{as}$ is fairly straightforward. In most countries, the VAT rate is applied on the VAT-exclusive retail price and amount-specific excise taxes are either volume-based (e.g., $0·10 per liter) or sugar-content-based (e.g., $0·10 per 10 grams of sugar).

On the other hand, calculating $A_{av}$ – the type of excise tax based on a percentage of the value of a beverage – is more challenging. For locally produced beverages, the base on which the rate is applied differs across countries, such that simply comparing reported statutory *ad valorem* excise tax rates without considering the base on which they apply would lead to biased results. As for VAT, it is fairly straightforward to estimate the tax base *B* in Eq. (2) when it is set in the latest stages of the value chain, such as the retail price, VAT-exclusive retail price, or VAT- and excise tax-exclusive retail price.

(2) $$S_{av}=\frac{A_{av}}{P}=\frac{T_{av}\times B}{P}$$

However, in cases where the tax base is determined earlier in the value chain, such as the producer price, as seen below in Eq. (3), where $T_{av}$ represents the *ad valorem* excise tax rate (in percentages), estimating the tax base *B* requires an assumption on the distribution margins π (retailer's and wholesaler's).

(3) $$B=\frac{P-A_{vat}-A_{as}-\pi }{\left(1+T_{av}\right)}$$

Country-specific information on distribution margins in the soft drink sector is rarely available. In France, they have been estimated at 47·2% on average among national grocery store chains.^30^ For the broader sector of food and non-alcoholic beverage, estimates from the United States (US) show an average gross margin of 28%,^31^^,^^32^ while representing 20% for small grocery stores in Mexico.^33^ In Australia, distribution margins were estimated at approximately 25%.^34^ Other studies investigating profit margins in this sector in LAC have mostly focused on the manufacturers’ margins. In Chile, one recent study found their gross profit margins to be 5–7% on average.^35^

For the tobacco tax share, WHO assumes distribution margins to be zero.^22^ While it could be assumed that retail margins are small for SSBs, assuming distribution margins to be zero would overestimate the base *B* and in turn the share of *ad valorem* excise taxes in the final retail price. On the other hand, there is a risk of underestimation by assuming high distribution margins in countries where the distribution of SSBs is a competitive market. Consequently, total distribution margins were assumed to represent a 20% mark-up. Applying this assumption to all countries using the producer price as tax base allows for standardized comparisons of excise tax share estimates among them. As we will show, this represents only a handful of countries. Additionally, it allows for fairer comparisons with countries using tax bases fixed later in the value chain by estimating a lower relative tax base for countries using the producer price. Sensitivity analysis for this assumption is discussed in the limitations section.

In the case of imported beverages, *ad valorem* excise taxes are typically applied on a base that includes the cost, insurance, and freight (CIF) value – defined as the value of the unloaded consignment that includes the cost of the product itself, insurance, and transport and unloading – and import and custom duties, when applicable. For import duties, rates were assumed lowest possible in case of preferential trade agreements and were typically applied on the CIF value.

Finally, other indirect taxes, such as custom service charges or environmental levies, were accounted for, when applicable. The latter are often applied based on beverage container type and were accounted for even when they were under the form of a deposit refunded if the container is returned, as they have an impact on the retail price faced by consumers.

### Data sources

Product information (volume size, sugar content, and country of origin), retail price data, and information on indirect taxes applied on non-alcoholic beverages – including structures, rates and bases, and tax administration information – were solicited directly from officially nominated Ministry of Finance practitioners through a survey conducted by PAHO regional and country offices between March and December 2019 (hereafter called PAHO SSB tax survey). This survey was completed by 27 PAHO Member States in LAC (all except Argentina, the Bahamas, the Plurinational State of Bolivia, Costa Rica, Haiti, and Nicaragua).

For tax information, we cross-checked the collected legislation with results from Sandoval et al.’s review of excise taxes on SSBs in LAC.^20^ For other indirect taxes, we reviewed legislation already collected through existing PAHO and WHO monitoring tools, including the WHO Global Nutrition Policy Review, the WHO Report on the Global Tobacco Epidemic, the WHO Global Information System on Alcohol and Health, and the PAHO NCD Country Capacity Survey, and conducted searches on websites of parliaments, ministries of finance, and legal databases. The data presented are based on legislation in effect as of 31 March 2019.

All retail prices are presented in international dollars (I$) at purchasing power parity (PPP) using the International Monetary Fund (IMF)’s implied PPP conversion rates for 2019.^36^ For countries for which tax rates were defined in US dollars (US$) rather than the local currency, we used the IMF's International Financial Statistics database exchange rates for March 2019.^37^ Finally, when CIF values were not provided by survey respondents, we estimated such values using the United Nations Comtrade database import statistics.^38^

### Beverages selected for the analysis

Due to the wide range of SSB categories consumed, it is impractical to collect data on all beverage categories over a large number of countries. For the PAHO SSB tax survey, sugar-sweetened carbonated drinks and fruit drinks – with less than 100% fruit concentration – were selected as they represent the two SSB categories with the highest market share in volume sold in LAC (no data available for most Caribbean countries).^39^^,^^40^ Energy drinks were also selected as volume sold has tripled in the last decade and they represent an emerging public health hazard, particularly for youths.^39^^,^^41^ The fourth and last SSB category included was sugar-sweetened milk drinks as they may promote increased free sugar and energy intake and a previous analysis found that the majority of countries in LAC do not apply excise taxes on this SSB category.^42^^,^^20^ Finally, bottled waters were included in order to capture differentiations between SSBs and non-sweetened beverages. Together, the five categories selected represent more than 90% of the market for non-alcoholic beverages in volume sold in LAC (no data available for most Caribbean countries).^39^

For sugar-sweetened carbonated drinks, regular Coca-Cola® was selected as an internationally comparable brand found in every country in LAC and the most sold in volume in the majority of countries.^39^ For the other beverage categories selected, no single brand was the most sold in the majority of countries in LAC, therefore each country was asked to select their most sold brand based on national market share information. Retail prices (inclusive of all indirect taxes, as applicable) were collected in hypermarkets/supermarkets and in convenience stores as the leading off-trade sales channels in LAC.^39^

Regarding beverage volume sizes, the most common size sold in Latin America (355 ml, no data available for most Caribbean countries) was selected for the internationally comparable brand of sugar-sweetened carbonated drinks.^43^ Due to a lack of market data, for bottled waters and energy drinks, survey respondents were requested to collect bottles sized for individual consumption, without specifying a particular volume size. Volume sizes were then standardized to 250 ml for energy drinks and 500 ml for bottled waters assuming a linear transformation of retail prices as they represented the respective modes of the distribution of volume sizes obtained. For the remaining beverages, data on 1000 ml bottles were collected to facilitate standardization as it represents the most common base for volume-based specific excise taxes. To assess differences in retail prices and tax shares by volume sizes, data on 1000 ml bottles of the internationally comparable brand of sugar-sweetened carbonated drinks were also collected. If the volume size requested for any beverage category was not available in a country, retail prices were adjusted to the selected standardized volume size assuming a linear transformation.

### Role of the funding source

The funding source had no role in the design of the study and collection, analysis, and interpretation of data or in writing the manuscript.

## Results

Table 1 contains retail price, excise tax share, and total tax share estimates for the beverages analyzed. See Tables S1–S6 in the *Supplementary material* for a more detailed presentation of the results for each beverage analyzed, including retail prices in local currency and US$, and excise taxes, VAT, import duties, and other taxes shares.

**Table 1:** Retail price, excise tax share, and total tax share for small and large sugar-sweetened carbonated drinks, fruit drinks, sugar-sweetened milk drinks, energy drinks, and bottled waters in Latin America and the Caribbean in 2019 (based on legislation in effect as of 31 March 2019).

	Sugar-sweetened carbonated drink, internationally comparable brand, small, 355 ml			Sugar-sweetened carbonated drink, internationally comparable brand, large, 1000 ml			Fruit drink, most sold brand, 1000 ml			Sugar-sweetened milk drink, most sold brand, 1000 ml			Energy drink, most sold brand, 250 ml			Bottled water, most sold brand, 500 ml		
Country	Retail price in PPP I$	Excise tax share	Total tax share	Retail price in PPP I$	Excise tax share	Total tax share	Retail price in PPP I$	Excise tax share	Total tax share	Retail price in PPP I$	Excise tax share	Total tax share	Retail price in PPP I$	Excise tax share	Total tax share	Retail price in PPP I$	Excise tax share	Total tax share
**Caribbean**																		
Antigua and Barbuda	1·24	0·0%	38·0%	2·43	0·0%	39·9%	2·89	0·0%	19·2%	4·03	0·0%	21·5%	1·97	0·0%	25·5%	0·48	0·0%	9·1%
Barbados	0·70	6·5%	21·4%	1·23	6·5%	21·4%	2·69	6·5%	21·4%	2·82	6·5%	21·4%	3·93	0·8%	17·2%	0·88	0·0%	17·8%
Belize	0·57	18·2%	29·3%	1·77	16·5%	27·6%	2·24	0·0%	11·1%	1·72	0·0%	12·5%	1·71	4·3%	34·1%	0·76	19·2%	30·4%
Dominica	1·04	4·0%	24·7%	. . .	. . .	. . .	4·01	0·0%	17·2%	16·11	0·0%	16·4%	1·46	0·7%	14·2%	1·12	0·0%	13·0%
Grenada	0·99	0·0%	13·0%	5·26	0·0%	13·0%	. . .	. . .	. . .	. . .	. . .	. . .	. . .	. . .	. . .	. . .	. . .	. . .
Guyana	1·19	0·0%	20·6%	2·58	0·0%	16·1%	3·90	0·0%	12·3%	7·58	0·0%	12·3%	1·91	0·0%	25·3%	0·54	0·0%	30·5%
Jamaica	0·71	0·0%	14·5%	1·52	0·0%	14·5%	7·89	0·0%	23·9%	9·18	0·0%	18·3%	0·78	0·0%	14·5%	0·64	0·0%	14·5%
Saint Kitts and Nevis	1·52	1·3%	2·9%	1·52	2·0%	4·3%	2·78	0·0%	3·8%	3·01	0·0%	4·1%	3·34	2·2%	11·5%	0·57	0·0%	0·0%
Saint Lucia	0·46	. . .	. . .	1·54	. . .	. . .	3·59	0·0%	13·9%	. . .	. . .	. . .	1·58	0·0%	12·8%	0·42	0·0%	11·1%
Saint Vincent and the Grenadines	1·77	4·3%	38·3%	3·77	5·6%	30·7%	4·03	0·0%	16·7%	3·77	. . .	. . .	1·84	8·6%	39·9%	1·62	0·0%	33·6%
Suriname	1·66	4·0%	9·8%	2·66	7·1%	16·2%	4·36	4·3%	13·4%	5·04	0·0%	0·0%	1·91	2·5%	18·7%	1·02	9·2%	9·2%
Trinidad and Tobago	0·90	0·0%	11·1%	1·19	0·0%	11·1%	2·39	0·0%	11·1%	5·82	0·0%	11·7%	1·87	0·0%	14·9%	0·73	0·0%	0·0%
**Latin America**																		
Brazil^a^	1·33	2·3%	29·7%	1·53	2·4%	27·8%	1·31	0·0%	0·0%	3·05	0·0%	0·0%	3·06	2·3%	31·1%	0·36	0·0%	40·1%
Chile	1·30	15·1%	31·1%	2·27	15·1%	31·1%	3·35	15·1%	31·1%	2·84	0·0%	16·0%	3·38	7·0%	23·0%	0·53	0·0%	16·0%
Colombia	1·36	0·0%	16·0%	1·53	0·0%	16·0%	1·70	0·0%	16·0%	7·61	0·0%	16·0%	1·01	0·0%	16·0%	1·09	0·0%	16·0%
Cuba	. . .	0·0%	9·9%	. . .	. . .	. . .	. . .	. . .	. . .	. . .	0·0%	42·0%	. . .	. . .	. . .	. . .	0·0%	42·0%
Dominican Republic	0·81	0·0%	15·3%	4·30	0·0%	15·3%	1·43	0·0%	15·3%	3·30	0·0%	15·3%	1·43	0·0%	15·3%	0·34	0·0%	0·0%
Ecuador	0·99	12·7%	27·2%	1·59	22·4%	35·5%	4·35	5·9%	16·6%	3·98	0·0%	10·7%	0·86	8·1%	23·3%	0·43	0·0%	19·6%
El Salvador	1·20	8·0%	19·5%	1·75	8·0%	19·5%	1·27	4·2%	15·7%	2·65	0·0%	11·5%	0·73	21·7%	33·2%	0·64	0·0%	11·5%
Guatemala	1·05	1·5%	12·3%	2·47	1·8%	12·6%	1·80	1·4%	13·0%	3·61	0·0%	10·7%	0·81	0·9%	12·2%	0·56	1·8%	12·5%
Honduras	1·01	2·6%	15·7%	1·64	4·5%	17·6%	2·06	3·6%	16·6%	2·80	0·0%	13·0%	3·24	0·6%	13·6%	0·76	0·0%	13·0%
Mexico	0·84	5·3%	19·1%	1·94	6·5%	20·3%	1·93	6·5%	6·5%	2·57	0·0%	0·0%	1·11	2·8%	16·6%	0·38	0·0%	0·0%
Panama	1·40	5·0%	5·0%	3·06	5·0%	5·0%	2·66	0·0%	0·0%	3·77	5·0%	5·0%	3·08	1·7%	1·7%	0·94	0·0%	0·0%
Paraguay	1·35	3·6%	12·7%	2·11	3·6%	12·7%	2·52	3·6%	12·7%	2·91	0·0%	9·1%	4·70	1·1%	13·7%	0·76	0·0%	9·1%
Peru	1·09	16·9%	32·2%	2·30	16·9%	32·2%	2·29	16·9%	32·2%	2·76	16·9%	32·2%	0·95	16·9%	32·2%	0·69	0·0%	15·3%
Uruguay	1·20	6·2%	24·3%	2·15	9·8%	27·8%	3·56	3·4%	21·4%	1·95	0·0%	18·0%	2·87	1·8%	19·9%	1·28	2·0%	20·0%
Venezuela (Bolivarian Republic of)	7·73	0·0%	13·8%	17·83	0·0%	13·8%	28·71	0·0%	13·8%	71·62	0·0%	13·8%	32·69	. . .	. . .	6·69	0·0%	13·8%

Source: Prepared by the authors using the study data

Notes:

Data only available for countries which completed PAHO SSB tax survey, i.e. all PAHO Member States in Latin America and the Caribbean except Argentina, the Bahamas, the Plurinational State of Bolivia, Costa Rica, Haiti, and Nicaragua.

The internationally comparable brand selected for sugar-sweetened carbonated drink is regular Coca-Cola®.

Retail price as faced by consumers (inclusive of all indirect taxes, as applicable).

. . .: No data available

ml: Milliliters

PPP I$: International dollars at purchasing power parity

^a^ Brazil: Retail price and tax data representing only the State of Rio de Janeiro. However, all indirect taxes applied on sugar-sweetened beverages in Brazil are applied at federal level, except the value added tax which rate varies by State.

**Figure 1:**
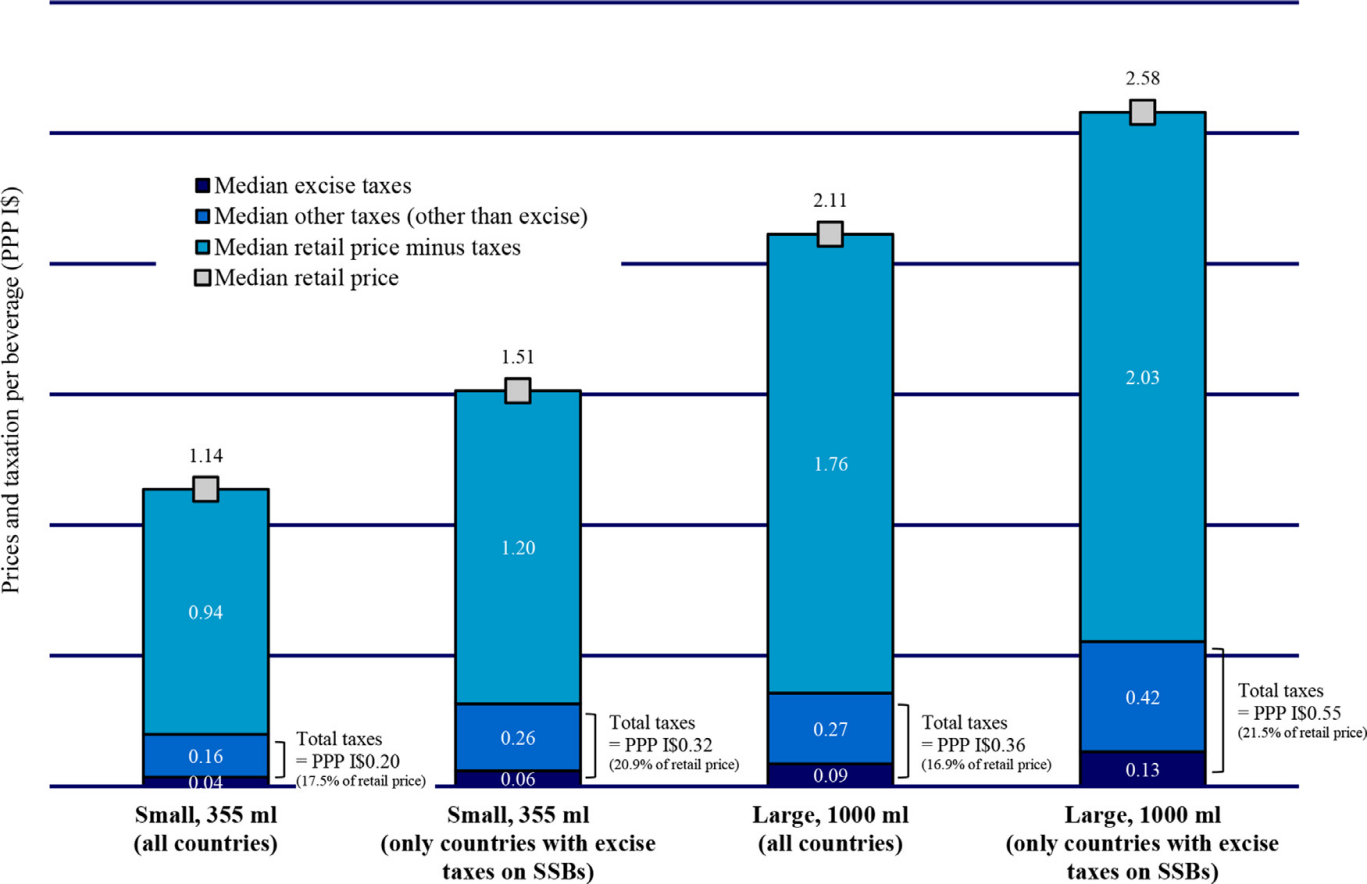
Median retail price and taxation on an internationally comparable brand of sugar-sweetened carbonated drink in Latin America and the Caribbean in 2019 (based on legislation in effect as of 31 March 2019). SSBs: Sugar-sweetened beverages. ml: Milliliters. PPP I$: International dollars at purchasing power parity. Retail price as faced by consumers (inclusive of all indirect taxes, as applicable). The internationally comparable brand selected for sugar-sweetened carbonated drink is regular Coca-Cola®. Data only available for countries which completed PAHO SSB tax survey, i.e. all PAHO Member States in Latin America and the Caribbean except Argentina, the Bahamas, the Plurinational State of Bolivia, Costa Rica, Haiti, and Nicaragua. Brazil: Retail price and tax data representing only the State of Rio de Janeiro. However, all indirect taxes applied on sugar-sweetened beverages in Brazil are applied at federal level, except the value added tax which rate varies by State. For small sugar-sweetened carbonated drink: No data available for Saint Lucia. For large sugar-sweetened carbonated drink: No data available for Cuba, Dominica, and Saint Lucia.Source: Prepared by the authors using the study data.

The median retail price of the internationally comparable brand of sugar-sweetened carbonated drink was PPP I$1·14 for 355 ml and PPP I$2·11 for 1000 ml (Figure 1), with retail prices ranging from PPP I$0·46 in Saint Lucia to PPP I$7·73 in the Bolivarian Republic of Venezuela for 355 ml and PPP I$1·19 in Trinidad and Tobago to PPP I$17·83 in the Bolivarian Republic of Venezuela for 1000 ml. If we take out the Bolivarian Republic of Venezuela, which represented an outlier due to its high 2019 PPP converter,^36^ the highest retail prices were found in Saint Vincent and the Grenadines and Grenada with PPP I$1·77 and PPP I$5·26, respectively, for small and large sugar-sweetened carbonated drinks (Table 1). Median retail prices were higher in countries applying excise taxes on SSBs than in countries that did not (Figure 1). The median retail price was slightly lower in the Caribbean than in Latin America (PPP I$1·01 vs. PPP I$1·20 for 355 ml and PPP I$1·77 vs. PPP I$2·13 for 1000 ml). Retail prices for the other beverages analyzed were less comparable between countries as no single most sold brand was collected. However, median retail prices for these beverages were higher in the Caribbean than in Latin America (fruit drinks: PPP I$3·59 vs. PPP I$2·18; sugar-sweetened milk drinks: PPP I$4·53 vs. PPP I$2·98; bottled waters: PPP I$0·73 vs. PPP I$0·66), except for energy drinks (PPP I$1·87 vs. PPP I$2·15).

**Figure 2:**
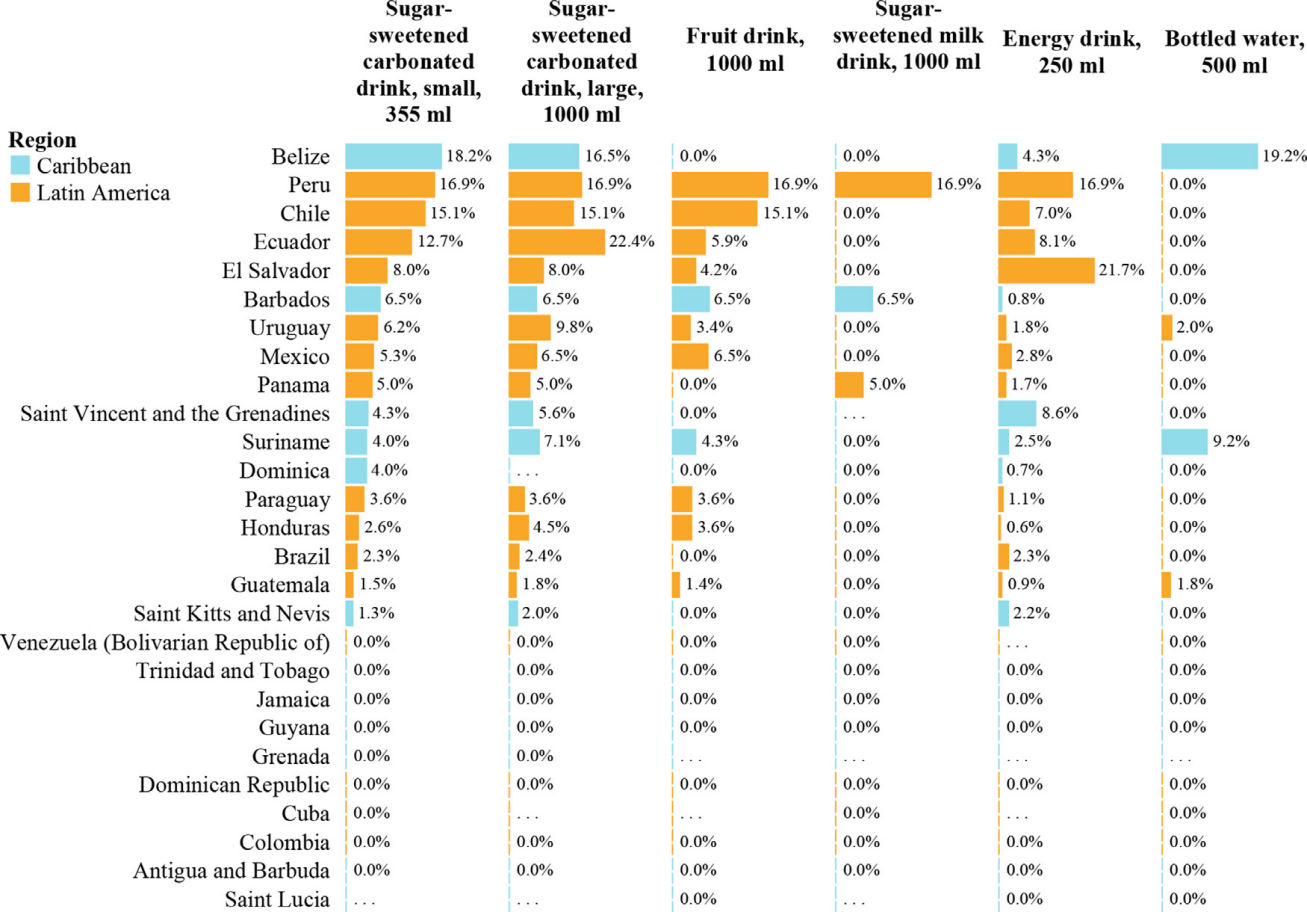
Excise tax share estimates for an internationally comparable brand of sugar-sweetened carbonated drink 355 ml and 1000 ml and the most sold brand of fruit drink 1000 ml, sugar-sweetened milk drink 1000 ml, energy drink 255 ml, and bottled water 500 ml, in Latin America and the Caribbean in 2019 (descending order ranking based on excise tax share on sugar-sweetened carbonated drink 355 ml, based on legislation in effect as of 31 March 2019). ml: Milliliters. Data only available for countries which completed PAHO SSB tax survey, i.e. all PAHO Member States in Latin America and the Caribbean except Argentina, the Bahamas, the Plurinational State of Bolivia, Costa Rica, Haiti, and Nicaragua. For sugar-sweetened carbonated drinks, an internationally comparable brand was selected. This was regular Coca-Cola®. For all other beverages included in this analysis, the respective most sold brand was selected. Brazil: Retail price and tax data representing only the State of Rio de Janeiro. However, all indirect taxes applied on sugar-sweetened beverages in Brazil are applied at federal level, except the value added tax which rate varies by State.Source: Prepared by the authors using the study data.

When including only countries applying excise taxes on these beverages, the highest median excise tax share was found for large sugar-sweetened carbonated drinks (6·5%) and sugar-sweetened milk drinks (6·5%, although only three of the countries analyzed applied excise taxes on this beverage category), followed by bottled waters (5·6%, although only four of the countries analyzed applied excise taxes on this beverage category), small sugar-sweetened carbonated drinks (5·0%), fruit drinks (4·3%), and finally energy drinks (2·3%). The highest excise tax share was found in El Salvador for energy drinks (21·7%), on which both *ad valorem* and volume-based specific excise taxes are applied. Out of the countries analyzed, 11 applied excise taxes on fruit drinks compared to 17 on sugar-sweetened carbonated and energy drinks. A higher proportion of Latin American countries applied excise taxes on SSBs than in the Caribbean, with higher excise tax shares also found in Latin America overall (Figure 2).

For small sugar-sweetened carbonated drinks, the median amount-specific excise tax share was slightly lower than the *ad valorem* excise tax share (4·7% vs. 5·0%), however the contrary was found for large sugar-sweetened carbonated drinks (7·1% vs. 5·7%). For *ad valorem* excise taxes, countries applying tax rates on a base set later in the value chain – closer to the final retail price – had higher *ad valorem* excise tax shares than countries using the producer price as base (6·5% vs. 3·6% and 6·8% vs. 3·6% for small and large sugar-sweetened carbonated drinks, respectively) (Tables S1–S6).

Regarding total tax share estimates, including all other indirect taxes as applicable, small sugar-sweetened carbonated drinks had a median of 17·5%, followed by large sugar-sweetened carbonated drinks and energy drinks (both 16·9%) (Figure 1), fruit drinks (15·3%), bottled waters (13·4%), and finally sugar-sweetened milk drinks (12·8%). The highest total tax share was found in Cuba for sugar-sweetened milk drinks and bottled waters (42·0%); however, the country only applies VAT on these beverages (Table 1). VAT were found to represent the main taxes applied on non-alcoholic beverages across LAC. Countries in the Caribbean were more likely to report imported most sold brands, which led to non-zero import and other custom duties, driving total tax share estimates up (Tables S1–S6).

Finally, we analyzed which countries earmark at least a portion of revenues from excise taxes on SSBs towards health programs. This complements the qualitative analysis by Sandoval et al.^20^ We found that this was the case in at least two countries, Mexico and Dominica (Table 2). In Mexico, the law stipulates that excise tax revenues should be earmarked towards the prevention and control of malnutrition, overweight, obesity and related NCDs, as well as increasing access to potable water. In Dominica, the law stipulates that excise tax revenues should be earmarked towards the national “Get Healthy” campaign.

**Table 2:** Information on the design of excise taxes on sugar-sweetened beverages in Latin America and the Caribbean (based on legislation in effect as of 31 March 2019).

Country	Excise tax structure*	Ad valorem tax base for locally produced beverages*	Automatic adjustment of amount-specific tax for inflation or other economic indicators*	Excise tax based on sugar content*	Uniform tax rate (No = Tiered)*	At least a portion of excise tax revenue is earmarked for health
Antigua and Barbuda	No excise	−	−	−	−	−
Argentina	Ad valorem	Retail price excluding VAT	−	No	No	. . .
Bahamas	No excise	−	−	−	−	−
Barbados	Ad valorem	Producer price	−	No	Yes	No
Belize	Amount-specific	−	No	No	Yes	No
Bolivia (Plurinational State of)	Amount-specific	−	Yes	No	No	. . .
Brazil	Ad valorem	Producer price	−	No	Yes	No
Chile	Ad valorem	Retail price excluding VAT	−	Yes^h^	No	No
Colombia	No excise	−	−	−	−	−
Costa Rica	Amount-specific	−	Yes	No	No	. . .
Cuba	No excise	−	−	−	−	−
Dominica	Combined^a^	Producer price	No	No	No	Yes
Dominican Republic	No excise	−	−	−	−	−
Ecuador	Combined^a^	Retail price excluding VAT and excise	Yes	Yes	No	No
El Salvador	Ad valorem^b^	Retail price excluding VAT and excise	No^f^	No	No	No
Grenada	No excise	−	−	−	−	−
Guatemala	Amount-specific	−	No	No	No	No
Guyana	No excise	−	−	−	−	−
Haiti	No excise^c^	−	−	−	−	−
Honduras	Amount-specific	−	Yes	No	Yes	No
Jamaica	No excise	−	−	−	−	−
Mexico	Amount-specific^b^	Producer price^e^	Yes	No	Yes	Yes
Nicaragua	Ad valorem	Retail price	−	No	No^i^	. . .
Panama	Ad valorem	Retail price	−	No	Yes	No
Paraguay	Ad valorem	Producer price	−	No	Yes	No
Peru	Ad valorem	Retail price excluding VAT and excise	−	Yes^h^	No	No
Saint Kitts and Nevis	Ad valorem	Retail price excluding VAT	−	No	Yes	No
Saint Lucia	No excise	−	−	−	−	−
Saint Vincent and the Grenadines	Ad valorem	Retail price excluding VAT	−	No	Yes	No
Suriname	Amount-specific	−	No	No	Yes	No
Trinidad and Tobago	No excise	−	−	−	−	−
Uruguay	Amount-specific^d^	Fixed tax base “precios fictos”	No^g^	No	No	No
Venezuela (Bolivarian Republic of)	No excise	−	−	−	−	−

Source: Prepared by the authors using data from Sandoval et al.^20^ and the study data.

Notes:

. . .: No data available

_: Not applicable

*: Data from Sandoval et al.^20^

^a^ Combined: At least one category of sugar-sweetened beverage is taxed by an *ad valorem* excise tax and at least one other category is taxed by an amount-specific excise tax. No beverage category is taxed by both. Dominica applies an *ad valorem* excise tax except for sugar-sweetened carbonates, which are subject to an amount-specific tax (volume-based). Ecuador imposes an amount-specific tax (sugar-content-based) on sugar-sweetened beverages with a sugar concentration above a specified threshold, and an *ad valorem* excise tax on SSBs below this threshold. All energy drinks (regardless of their sugar concentration) are taxed by the *ad valorem* tax.

^b^ In El Salvador and Mexico, energy drinks are subject to a mixed excise tax system, i.e. taxed by both an *ad valorem* and an amount-specific component.

^c^ Haiti: The country did not participate in PAHO SSB tax survey in 2019. A law from 1971, “Loi sur le Droit d'Accise du 21 Octobre 1971,” imposes an amount-specific excise tax both on imported and locally produced carbonated drinks. However, a World Trade Organization report states that as of June 2015, the excise tax had a different structure for imported (amount-specific) and locally produced (*ad valorem*) carbonated drinks, which could constitute a violation of national treatment.^44^ We did not find more recent information or legislation regarding this tax. Due to the potential discriminatory nature of the tax between imported and locally produced beverages and the lack of information, this tax was not included in the analysis.

^d^ Uruguay: The excise tax is structured as an *ad valorem* tax applied on fixed tax base amounts – “precios fictos” – per volume varying per beverage category, effectively operating as an amount-specific tax (volume-based) and classified as such in this analysis.

^e^ Mexico: The *ad valorem* component applies only to energy drinks. In 2019, it was applied only on energy drinks with more than 20 mg of caffeine per 100 ml. This threshold was eliminated in 2020 by the law “Ley de Ingresos de la Federación para el Ejercicio Fiscal de 2020”, and the *ad valorem* component is now applied on all energy drinks.

^f^ El Salvador: The amount-specific component applies only to energy drinks.

^g^ Uruguay: The fixed tax base amounts – “precios fictos” – are usually adjusted annually; however, it is not mandated by law.

^h^ Chile and Peru: Tiered design with different *ad valorem* tax rates defined by sugar concentration thresholds.

^i^ Nicaragua: The *ad valorem* tax rate is uniform for sugar-sweetened beverages, but a lower rate applies to mineral water.

## Discussion

The results highlight that as of March 2019, 12 countries did not apply excise taxes on SSBs in LAC and most countries apply low excise taxes – representing less than 10% of the final retail price. Excise taxes on SSBs are mostly lower in the Caribbean than in Latin America, which is concerning given the fact that the Caribbean has the highest average daily adult SSB consumption in the world.^1^

WHO recommends that excise taxes should increase the retail price of SSBs (as faced by consumers, including all indirect taxes, as applicable) by at least 20% to result in significant reductions in consumption.^45^ While this does not represent a recommendation on a minimum excise tax level – as its impact on retail prices is dependent on the degree of passthrough of the tax in each country – it can be assimilated to a 16·6% excise tax share for the purpose of this analysis, assuming a full passthrough. Notably, we found that only two countries applied excise taxes on sugar-sweetened carbonated drinks (the most consumed SSB category) at or above this threshold (Belize and Peru for smaller size drinks, and Ecuador and Peru for larger size drinks). Despite being introduced with an explicit health rationale, the SSB excise taxes in Barbados and Mexico fall significantly below this threshold with an estimated excise tax share of 6·5% and 5·3%, respectively for small sugar-sweetened carbonated drinks.

As expected, we found that amount-specific excise tax shares increase with beverage volume sizes. As the container size of a beverage increases, volume-based specific excise taxes increase and the retail price per milliliter decreases. Therefore, quantity discounts are taxed, which is not the case with *ad valorem* excise taxes. In addition, *ad valorem* excise taxes have more variable impacts on retail prices; as seen in the results of our analysis, *ad valorem* taxes applied on a value set early in the value chain have a smaller impact on retail prices than if applied based on the retail price. These advantages, among others, support the general recommendation to focus on amount-specific rather than *ad valorem* excise taxes.^18^

Many excise taxes applied on SSBs exhibit a certain degree of policy incoherence from a health point of view, as their design fails to effectively create a tax differential between SSBs and non-sweetened beverages. For example, many countries do not apply such taxes on fruit drinks and only a few do so on sugar-sweetened milk drinks and liquid and powder concentrates used to make SSBs.^20^ Also, some countries apply excise taxes on bottled waters, with excise tax shares often higher than for small sugar-sweetened carbonated drinks, fruit drinks, and energy drinks (e.g., Belize, Suriname). This undermines the ability of these taxes to generate a price differential to incentivize consumers to switch from consuming SSBs to a healthier alternative. In addition, some excise taxes are designed with different rates based on the definition of SSB categories. This may create opportunities for substitutions that are not in line with public health goals of lowered absolute free sugars consumption, for example, if some SSBs with higher sugar concentration are taxed at lower rates or not taxed. In countries with a sufficiently strong tax administration, it may be best to apply excise taxes based on sugar content – either through volume-based specific or *ad valorem* excise taxes tiered by sugar concentration thresholds, or through sugar-content-based specific excise taxes – so that all SSBs are taxed and those with higher sugar concentration are taxed at higher rates.^18^

VAT represent the main component of total tax share estimates. While such taxes participate in increasing SSB retail prices, they also apply on most other products in the economy (including non-SSBs). Import and other custom duties can also represent a significant proportion of total taxes applied on some imported SSBs. However, because domestically produced substitutes are available in most countries, such taxes may lead to negative tax-induced substitutions towards locally produced SSBs. Therefore, unlike excise taxes, VAT and import and custom duties are not considered effective policy tools to change the relative price and lower the consumption of SSBs.^18^

Lastly, we only found evidence of two countries earmarking at least a portion of excise tax revenues from SSBs for health purposes, even though nine countries do so in LAC for tobacco excise taxes.^22^ Using soft earmarking of some portion of excise tax revenue for specific government programs toward health promotion or other public goods may help to garner public support for an SSB tax while complementing its intended health impact.^46^ An example of potential related programs that could be supported by excise tax revenues from SSBs is subsidizing drinking water infrastructure, as 35% of the population in LAC still does not have access to safe drinking water.^47^

### Policy implications

This analysis provides the first region-wide estimation of a standardized and comparable metric of tax levels applied on SSBs. PAHO's method only requires tax design information and nominal retail price data and enables comparisons of tax levels across beverage categories and countries with different tax designs, otherwise not comparable based solely on their statutory definition (Table 3).^29^

**Table 3:** Comparing statutory excise tax rates and excise tax share estimates for an internationally comparable brand of sugar-sweetened carbonated drink, 355 ml, for selected countries in Latin America and the Caribbean in 2019 (based on legislation in effect as of 31 March 2019).

Country	Statutory excise tax structure and rate on sugar-sweetened carbonated drinks as defined in the legislation	Excise tax share estimate for sugar-sweetened carbonated drink, internationally comparable brand, 355 ml (%)
Barbados	*Ad valorem* – 10% of the producer price	6·5%
Chile	*Ad valorem* – 18% of the retail price excluding VAT if sugar content > 15 g per 240 ml Otherwise, 10%	15·1%
Ecuador	Amount-specific (sugar-content based) – USD 0·18 per 100 g of sugars if sugar content > 25 g per liter Otherwise, *ad valorem* – 10% of the retail price excluding VAT and excise	12·7%
Mexico	Amount-specific (volume-based) – MXN 1·17 per liter	5·3%

Source: Prepared by the authors using the study data

Notes:

The internationally comparable brand selected for sugar-sweetened carbonated drink is regular Coca-Cola®.

ml: Milliliters

g: Grams

VAT: Value added tax

USD: United States Dollar

MXN: Mexican Peso

More than a decade of monitoring tobacco taxes using WHO's tobacco tax share indicator has proven that such indicator can inform excise tax policy design and institutional opportunities or barriers to apply such taxes. When presented using tax share leader boards (Figures. 2, and S1), it can also represent a powerful tool to advocate for the implementation, design improvement, or increase of excise taxes, especially with ministries of finance.^21^

The median excise tax share estimates for SSBs (2·3–6·5%, if only countries with excise taxes on SSBs are taken into account) are significantly lower than the median excise tax share for cigarettes in LAC (36·7% in 2020).^22^ WHO recommends that excise taxes represent at least 70% of the final retail price of cigarettes.^48^ As discussed previously, the current WHO recommendation for excise taxes on SSBs to increase retail prices by at least 20% requires an assumption on the tax passthrough in each country. Therefore, unlike the minimum recommended tobacco excise tax share, it does not provide a standardized minimum threshold. If scaled globally, the estimation of the tax share indicator for SSBs could inform the future formulation of a recommended minimum level of excise taxes as a percentage of the retail price.

Finally, as our analysis shows, there is room for higher and structurally improved excise taxes on SSBs in LAC. This could contribute to preventing obesity and other NCDs by reducing the consumption of SSBs and to raising additional and immediate tax revenue. This is especially needed in light of the current COVID-19 pandemic.^49^, ^50^, ^51^

### Limitations

In some countries, national market share data were not available, and PAHO SSB tax survey respondents were asked to consult vendors to select the most sold brand. This potentially led to the selection of most sold brands that may not be nationally representative. In addition, survey respondents were mostly based in the capital city of their respective country; thus, collected retail prices may also not be nationally representative. Only hypermarkets/supermarkets data – representing the second off-trade sales channel in volume sold –^39^ were used in our analysis due to a significant level of missing retail price data for convenience stores in the survey results. Where such prices were collected, they were usually found to be higher than hypermarkets/supermarkets prices and could potentially have led to slightly lower tax share estimates.

While the PAHO SSB tax survey explicitly requested information on fruit drinks with less than 100% juice concentration, the broad definition of harmonized tariff code 2009 – which includes fruit juices “whether or not containing added sugar or other sweetening matter” –^52^ may have led to errors in product and brand selection. Although 100% fruit juices and liquid and powder concentrates are SSBs, and as such should be subject to taxation, given the high heterogeneity of products within these categories and across countries, it was decided to exclude them from our analysis to preserve greater comparability. In addition, they represent a relatively small market share in LAC (0·5% and 0·6% of non-alcoholic beverage in volume sold, respectively, excluding hot drinks, no data available for most Caribbean countries).^43^

As seen in our results, as the container size of a beverage increases the retail price per milliliter of this beverage decreases. Therefore, linearly transforming retail prices to selected standardized volume sizes, as done in our analysis, may alter tax share estimations. However, for each beverage category, the mode of the distribution of volume sizes collected was found to be equal to the respective standardized volume size selected for our analysis, which minimized the number of linear transformations of retail prices required.

There may be concerns about our arbitrary 20% total distribution margins mark-up assumption as it could have led us to biased tax share estimates for countries using the producer price as *ad valorem* excise tax base for locally produced beverages. However, this assumption was only used for three countries in our sample (Barbados, Brazil, and Dominica). In these countries, total and excise tax share estimates were only slightly sensitive to different levels of distribution margins mark-up assumption, varying, respectively by a maximum of ± 3.6 and ± 2.4 percentage points, in absolute terms, when changing the mark-up assumption to different values between 0% and 50% – a broad range encompassing the distribution margin estimates found in the literature (Table S7).

Finally, data presented in our analysis are based on tax legislation in effect as of 31 March 2019. Legislation that could have been replaced, amended, or repealed since this cutoff date were not analyzed to maintain comparability of data at the same point in time across countries.

### Future research needs

Evidence has shown that consumers may substitute from taxed beverages to untaxed beverages or between taxed beverages following tax increases.^16^^,^^53^ Our analysis could therefore benefit from including other categories of SSBs, such as fruit juices or liquid and powder concentrates. In addition, it is important that future research analyzes the price dispersion in each SSB category, as emerging evidence has shown the potential for consumers to substitute for cheaper brands following a tax increase.^17^

It is necessary to develop global, periodic, and standardized monitoring systems to capture changes in consumption, affordability, tax designs, and levels of taxation applied on SSBs to allow comparisons over time and across countries. There is an institutional opportunity for WHO to do so building on the already existing tobacco tax monitoring framework and this paper.^21^ This could particularly inform the establishment of best practices in tax design.

Finally, while standardized tax share estimates are important to inform the discussion on a minimum recommended level of excise taxes on SSBs, the debate would benefit from additional data on excise tax revenue derived from SSBs and estimates of the economic cost of diseases attributable to their consumption.^54^ Recent evidence in LAC shows that such costs are significant.^55^

## Conclusions

We used PAHO's pragmatic approach – derived from WHO's well-established method for monitoring tobacco taxes – to estimate standardized and comparable tax share estimates for a range of SSB categories in LAC. We show that, although most LAC countries apply excise taxes on SSBs, tax levels remain low. This is true even in some countries which have pioneered the use of SSB taxes and received significant attention from the media and researchers (e.g., Barbados, Mexico). Our results also exhibit policy incoherence from a public health point of view and suboptimal tax designs in many cases (e.g., SSBs which are untaxed or bottled waters which are taxed), highlighting opportunities for reforms to increase tax rates and improve tax designs to increase the impact of existing excise taxes on SSB consumption and health.

Tax share estimates represent a powerful tool to compare levels of taxation across countries and beverage categories, as well as monitor trends over time, especially when interpreted alongside measures of affordability and consumption. They can be used to empirically assess the impact of a variety of tax designs, further guiding the development of SSB taxation best practice. Based on the tobacco tax share experience, we suggest that applying this study's approach more widely – in terms of geography and SSB categories considered – and over successive years would enable policy makers to optimize the use of SSB taxes and generate additional political will and attention around them. This could be done expanding WHO's monitoring framework for tobacco taxation to SSBs.

## Contributors

RCS, FSG, and MR conceptualized the study. All authors contributed to the design and implementation of data collection processes and tools. MR and MA analyzed the data and interpreted the results with input from all authors. MR and MA drafted the manuscript and all authors critically revised it. All authors reviewed and approved the final version.

## Data sharing statement

The nominal retail price data collected through the PAHO SSB tax survey is available in the supplementary material. Tax information was derived from publicly available national legislation documents. A detailed description of the study design and methods is available (https://iris.paho.org/handle/10665.2/54917).

## Funding

Bloomberg Philanthropies has provided funds for a grant agreement between the Pan American Health Organization and the Global Health Advocacy Incubator on obesity prevention policies.

## Declaration of interests

The authors have no conflicts of interest to disclose.

RCS and FSG are staff members of PAHO/WHO. Authors hold sole responsibility for the views expressed in the manuscript, which may not necessarily reflect the opinion or policy of PAHO/WHO.

## Appendix Supplementary materials

Image, application 1

Image, application 2
