# Supplementary material for: Comparing taxes as a percentage of sugar-sweetened beverage prices in Latin America and the Caribbean
Source: Lancet Reg Health Am. 2022 Apr 21;11:100257. doi: 10.1016/j.lana.2022.100257 (PMC9290324; doi:10.1016/j.lana.2022.100257)
Supplement: Supplementary file 1 [file mmc1.pdf]

Supplementary material

Supplemental material for: Roche M, Alvarado M, Sandoval RC, Gomes FS, Paraje G. Comparing taxes as a percentage of sugar-sweetened beverage prices in Latin America and the Caribbean.

Table of contents: Supplemental material for online publication

Table S1. Retail price and detailed tax share estimates for an international brand of sugar-sweetened carbonated drink, small, 355 ml, in Latin America and the Caribbean in 2019 (based on legislation in effect as of 31 March 2019)....1

Table S2. Retail price and detailed tax share estimates for an international brand of sugar-sweetened carbonated drink, large, 1000 ml, in Latin America and the Caribbean in 2019 (based on legislation in effect as of 31 March 2019)....3

Table S3. Retail price and detailed tax share estimates for the most sold brand of fruit drink, 1000 ml, in Latin America and the Caribbean in 2019 (based on legislation in effect as of 31 March 2019) 5

Table S4. Retail price and detailed tax share estimates for the most sold brand of sugar-sweetened milk drink, 1000 ml, in Latin America and the Caribbean in 2019 (based on legislation in effect as of 31 March 2019)....7

Table S5. Retail price and detailed tax share estimates for the most sold brand of energy drink, 1000 ml, in Latin America and the Caribbean in 2019 (based on legislation in effect as of 31 March 2019) 9

Table S6. Retail price and detailed tax share estimates for the most sold brand of water bottle, 500 ml, in Latin America and the Caribbean in 2019 (based on legislation in effect as of 31 March 2019) 11

Figure S1. Descending order ranking of decomposed total tax share estimates for an internationally comparable brand of sugar-sweetened carbonated drink, small, 355ml, in Latin America and the Caribbean in 2019 (based on legislation in effect as of 31 March 2019) 13

Table S7. Sensitivity of excise and total tax share estimates for ad valorem excise taxes using producer price as tax base for locally produced sugar-sweetened beverages and the distribution margins mark-up assumption 14

Table S1. Retail price and detailed tax share estimates for an international brand of sugar-sweetened carbonated drink, small, 355 ml, in Latin America and the Caribbean in 2019 (based on legislation in effect as of 31 March 2019)

	Retail price of an internationally comparable brand of sugar-sweetened carbonated drink, small, 355 ml				Taxes as a percentage of retail price					
Country	Retail price in local currency	Local currency[b]	Retail price in international dollar (PPP)	Retail price in US dollar	Amount-specific excise	Ad valorem excise	Value-added/sales taxes	Import duties	Other taxes	Total tax
Antigua and Barbuda	2.55	XCD	1.24	0.94	0.00%	0.00%	13.04%	10.13%	14.87%	38.0%
Barbados	1.55	BBD	0.70	0.78	0.00%	6.55%	14.89%	0.00%	0.00%	21.4%
Belize	0.75	BZD	0.57	0.38	18.22%	0.00%	11.11%	0.00%	0.00%	29.3%
Brazil[a]	3.04	BRL	1.33	0.78	0.00%	2.34%	19.70%	0.00%	7.62%	29.7%
Chile	534.00	CLP	1.30	0.78	0.00%	15.13%	15.97%	0.00%	0.00%	31.1%
Colombia	1 863.75	COP	1.36	0.58	0.00%	0.00%	15.97%	0.00%	0.00%	16.0%
Cuba	25.00	CUP	. . .	. . .	0.00%	0.00%	0.00%	9.90%	0.00%	9.9%
Dominica	1.78	XCD	1.04	0.66	4.00%	0.00%	13.04%	5.97%	1.64%	24.7%
Dominican Republic	18.02	DOP	0.81	0.36	0.00%	0.00%	15.25%	0.00%	0.00%	15.3%
Ecuador	0.52	USD	0.99	0.52	12.68%	0.00%	10.71%	0.00%	3.84%	27.2%
El Salvador	0.55	USD	1.20	0.55	0.00%	8.05%	11.50%	0.00%	0.00%	19.5%
Grenada	1.62	XCD	0.99	0.60	0.00%	0.00%	13.04%	0.00%	0.00%	13.0%
Guatemala	4.14	GTQ	1.05	0.54	1.54%	0.00%	10.71%	0.00%	0.00%	12.3%
Guyana	120.00	GYD	1.19	0.58	0.00%	0.00%	12.28%	0.00%	8.33%	20.6%
Honduras	10.65	HNL	1.01	0.44	2.61%	0.00%	13.04%	0.00%	0.00%	15.7%
Jamaica	50.00	JMD	0.71	0.40	0.00%	0.00%	14.16%	0.00%	0.32%	14.5%
Mexico	7.81	MXN	0.84	0.40	5.32%	0.00%	13.79%	0.00%	0.00%	19.1%
Panama	0.67	PAB	1.40	0.67	0.00%	5.00%	0.00%	0.00%	0.00%	5.0%
Paraguay	3 443.50	PYG	1.35	0.56	0.00%	3.64%	9.09%	0.00%	0.00%	12.7%
Peru	1.89	PEN	1.09	0.57	0.00%	16.95%	15.25%	0.00%	0.00%	32.2%
Saint Kitts and Nevis	3.00	XCD	1.52	1.11	0.00%	1.35%	0.00%	0.00%	1.53%	2.9%
Saint Lucia	0.89	XCD	0.46	. . .	. . .	. . .	. . .	. . .	. . .	. . .
Saint Vincent and the Grenadines	2.75	XCD	1.77	1.02	0.00%	4.27%	13.79%	0.00%	20.21%	38.3%
Suriname	4.56	SRD	1.66	0.61	4.04%	0.00%	5.73%	0.00%	0.00%	9.8%
Trinidad and Tobago	3.75	TTD	0.90	0.56	0.00%	0.00%	11.11%	0.00%	0.00%	11.1%
Uruguay	31.24	UYU	1.20	0.93	6.24%	0.00%	18.03%	0.00%	0.00%	24.3%
Venezuela (Bolivarian Republic of)	5 200.00	VES	7.73	. . .	0.00%	0.00%	13.79%	0.00%	0.00%	13.8%

Source: Prepared by the authors using the study data.

Notes:

The internationally comparable brand selected for sugar-sweetened carbonated drinks is regular Coca-Cola®.

. . .: No data available

ml: Milliliters

PPP: Purchasing power parity

Data only available for countries which completed PAHO SSB tax survey, i.e. all PAHO Member States in Latin America and the Caribbean except Argentina, the Bahamas, the Plurinational State of Bolivia, Costa Rica, Haiti, and Nicaragua.

[a] Brazil: Retail price and tax data representing only the State of Rio de Janeiro. However, all indirect taxes applied on sugar-sweetened beverages in Brazil are applied at federal level, except the value added tax which rate varies by State.

[b] Local currency codes: According to International Organization for Standardization, ISO 4217 currency names and code elements (https://www.iso.org/iso-4217-currency-codes.html).

Table S2. Retail price and detailed tax share estimates for an international brand of sugar-sweetened carbonated drink, large, 1000 ml, in Latin America and the Caribbean in 2019 (based on legislation in effect as of 31 March 2019)

Country	Retail price of an internationally comparable brand of sugar-sweetened carbonated drink, large, 1000 ml				Taxes as a percentage of retail price					
	Retail price in local currency	Local currency[b]	Retail price in international dollar (PPP)	Retail price in US dollar	Amount-specific excise	Ad valorem excise	Value-added/sales taxes	Import duties	Other taxes	Total tax
Antigua and Barbuda	5.00	XCD	2.43	1.85	0.00%	0.00%	13.04%	14.56%	12.29%	39.9%
Barbados	2.75	BBD	1.23	1.37	0.00%	6.55%	14.89%	0.00%	0.00%	21.4%
Belize	2.33	BZD	1.77	1.17	16.50%	0.00%	11.11%	0.00%	0.00%	27.6%
Brazil[a]	3.50	BRL	1.53	0.90	0.00%	2.41%	17.61%	0.00%	7.82%	27.8%
Chile	932.67	CLP	2.27	1.37	0.00%	15.13%	15.97%	0.00%	0.00%	31.1%
Colombia	2 100.00	COP	1.53	0.66	0.00%	0.00%	15.97%	0.00%	0.00%	16.0%
Cuba	. . .	CUP	. . .	. . .	. . .	. . .	. . .	. . .	. . .	. . .
Dominica	. . .	XCD	. . .	. . .	. . .	. . .	. . .	. . .	. . .	. . .
Dominican Republic	95.14	DOP	4.30	1.88	0.00%	0.00%	15.25%	0.00%	0.00%	15.3%
Ecuador	0.84	USD	1.59	0.84	22.40%	0.00%	10.71%	0.00%	2.39%	35.5%
El Salvador	0.80	USD	1.75	0.80	0.00%	8.05%	11.50%	0.00%	0.00%	19.5%
Grenada	8.60	XCD	5.26	3.19	0.00%	0.00%	13.04%	0.00%	0.00%	13.0%
Guatemala	9.78	GTQ	2.47	1.27	1.84%	0.00%	10.71%	0.00%	0.00%	12.6%
Guyana	261.00	GYD	2.58	1.25	0.00%	0.00%	12.28%	0.00%	3.83%	16.1%
Honduras	17.33	HNL	1.64	0.71	4.52%	0.00%	13.04%	0.00%	0.00%	17.6%
Jamaica	106.50	JMD	1.52	0.85	0.00%	0.00%	14.16%	0.00%	0.32%	14.5%
Mexico	18.00	MXN	1.94	0.93	6.50%	0.00%	13.79%	0.00%	0.00%	20.3%
Panama	1.47	PAB	3.06	1.47	0.00%	5.00%	0.00%	0.00%	0.00%	5.0%
Paraguay	5 400.00	PYG	2.11	0.87	0.00%	3.64%	9.09%	0.00%	0.00%	12.7%
Peru	4.00	PEN	2.30	1.21	0.00%	16.95%	15.25%	0.00%	0.00%	32.2%
Saint Kitts and Nevis	3.00	XCD	1.52	1.11	0.00%	2.02%	0.00%	0.00%	2.29%	4.3%
Saint Lucia	3.00	XCD	1.54	. . .	. . .	. . .	. . .	. . .	. . .	. . .
Saint Vincent and the Grenadines	5.85	XCD	3.77	2.17	0.00%	5.65%	13.79%	0.00%	11.24%	30.7%
Suriname	7.33	SRD	2.66	0.98	7.08%	0.00%	9.09%	0.00%	0.00%	16.2%
Trinidad and Tobago	4.98	TTD	1.19	0.74	0.00%	0.00%	11.11%	0.00%	0.00%	11.1%
Uruguay	56.00	UYU	2.15	1.67	9.80%	0.00%	18.03%	0.00%	0.00%	27.8%
Venezuela (Bolivarian Republic of)	12 000.00	VES	17.83	. . .	0.00%	0.00%	13.79%	0.00%	0.00%	13.8%

Source: Prepared by the authors using the study data.

Notes:
The internationally comparable brand selected for sugar-sweetened carbonated drinks is regular Coca-Cola®.
. . .: No data available
ml: Milliliters
PPP: Purchasing power parity
Data only available for countries which completed PAHO SSB tax survey, i.e. all PAHO Member States in Latin America and the Caribbean except Argentina, the Bahamas, the Plurinational State of Bolivia, Costa Rica, Haiti, and Nicaragua.
[a] Brazil: Retail price and tax data representing only the State of Rio de Janeiro. However, all indirect taxes applied on sugar-sweetened beverages in Brazil are applied at federal level, except the value added tax which rate varies by State.
[b] Local currency codes: According to International Organization for Standardization, ISO 4217 currency names and code elements (https://www.iso.org/iso-4217-currency-codes.html).

Table S3. Retail price and detailed tax share estimates for the most sold brand of fruit drink, 1000 ml, in Latin America and the Caribbean in 2019 (based on legislation in effect as of 31 March 2019)

Country	Retail price of a fruit drink of the most sold brand, 1000 ml				Taxes as a percentage of retail price					
	Retail price in local currency	Local currency[b]	Retail price in international dollar (PPP)	Retail price in US dollar	Amount-specific excise	Ad valorem excise	Value-added/sales taxes	Import duties	Other taxes	Total tax
Antigua and Barbuda	5.95	XCD	2.89	2.20	0.00%	0.00%	13.04%	0.00%	6.17%	19.2%
Barbados	5.99	BBD	2.69	3.00	0.00%	6.55%	14.89%	0.00%	0.00%	21.4%
Belize	2.95	BZD	2.24	1.48	0.00%	0.00%	11.11%	0.00%	0.00%	11.1%
Brazil[a]	2.99	BRL	1.31	0.77	0.00%	0.00%	0.00%	0.00%	0.00%	0.0%
Chile	1 379.33	CLP	3.35	. . .	0.00%	15.13%	15.97%	0.00%	0.00%	31.1%
Colombia	2 330.00	COP	1.70	0.73	0.00%	0.00%	15.97%	0.00%	0.00%	16.0%
Cuba	. . .	CUP	. . .	. . .	. . .	. . .	. . .	. . .	. . .	. . .
Dominica	6.86	XCD	4.01	2.54	0.00%	0.00%	13.04%	0.00%	4.13%	17.2%
Dominican Republic	31.71	DOP	1.43	0.63	0.00%	0.00%	15.25%	0.00%	0.00%	15.3%
Ecuador	2.28	USD	4.35	2.28	5.91%	0.00%	10.71%	0.00%	0.00%	16.6%
El Salvador	0.58	USD	1.27	0.58	0.00%	4.21%	11.50%	0.00%	0.00%	15.7%
Grenada	. . .	XCD	. . .	. . .	. . .	. . .	. . .	. . .	. . .	. . .
Guatemala	7.10	GTQ	1.80	0.92	1.41%	0.00%	10.71%	0.93%	0.00%	13.0%
Guyana	394.00	GYD	3.90	1.89	0.00%	0.00%	12.28%	0.00%	0.00%	12.3%
Honduras	21.78	HNL	2.06	0.89	3.60%	0.00%	13.04%	0.00%	0.00%	16.6%
Jamaica	553.90	JMD	7.89	4.43	0.00%	0.00%	17.70%	5.76%	0.49%	23.9%
Mexico	17.94	MXN	1.93	0.93	6.52%	0.00%	0.00%	0.00%	0.00%	6.5%
Panama	1.28	PAB	2.66	1.28	0.00%	0.00%	0.00%	0.00%	0.00%	0.0%
Paraguay	6 450.00	PYG	2.52	1.04	0.00%	3.64%	9.09%	0.00%	0.00%	12.7%
Peru	3.99	PEN	2.29	1.20	0.00%	16.95%	15.25%	0.00%	0.00%	32.2%
Saint Kitts and Nevis	5.50	XCD	2.78	2.04	0.00%	0.00%	0.00%	0.00%	3.79%	3.8%
Saint Lucia	6.99	XCD	3.59	2.59	0.00%	0.00%	11.11%	0.00%	2.78%	13.9%
Saint Vincent and the Grenadines	6.25	XCD	4.03	2.31	0.00%	0.00%	13.79%	0.00%	2.86%	16.7%
Suriname	12.00	SRD	4.36	1.61	4.33%	0.00%	9.09%	0.00%	0.00%	13.4%
Trinidad and Tobago	9.99	TTD	2.39	1.48	0.00%	0.00%	11.11%	0.00%	0.00%	11.1%
Uruguay	93.00	UYU	3.56	2.78	3.39%	0.00%	18.03%	0.00%	0.00%	21.4%
Venezuela (Bolivarian Republic of)	19 323.33	VES	28.71	. . .	0.00%	0.00%	13.79%	0.00%	0.00%	13.8%

Source: Prepared by the authors using the study data.

Notes:
. . .: No data available
ml: Milliliters
PPP: Purchasing power parity
Data only available for countries which completed PAHO SSB tax survey, i.e. all PAHO Member States in Latin America and the Caribbean except Argentina, the Bahamas, the Plurinational State of Bolivia, Costa Rica, Haiti, and Nicaragua.
[a] Brazil: Retail price and tax data representing only the State of Rio de Janeiro. However, all indirect taxes applied on sugar-sweetened beverages in Brazil are applied at federal level, except the value added tax which rate varies by State.
[b] Local currency codes: According to International Organization for Standardization, ISO 4217 currency names and code elements (https://www.iso.org/iso-4217-currency-codes.html).

Table S4. Retail price and detailed tax share estimates for the most sold brand of sugar-sweetened milk drink, 1000 ml, in Latin America and the Caribbean in 2019 (based on legislation in effect as of 31 March 2019)

Country	Retail price of a sugar-sweetened milk drink of the most sold brand, 250 ml				Taxes as a percentage of retail price					
	Retail price in local currency	Local currency[b]	Retail price in international dollar (PPP)	Retail price in US dollar	Amount-specific excise	Ad valorem excise	Value-added/sales taxes	Import duties	Other taxes	Total tax
Antigua and Barbuda	8.28	XCD	4.03	3.07	0.00%	0.00%	13.04%	0.00%	8.48%	21.5%
Barbados	6.29	BBD	2.82	3.15	0.00%	6.55%	14.89%	0.00%	0.00%	21.4%
Belize	2.27	BZD	1.72	1.14	0.00%	0.00%	11.11%	0.00%	1.38%	12.5%
Brazil[a]	6.95	BRL	3.05	1.78	0.00%	0.00%	0.00%	0.00%	0.00%	0.0%
Chile	1 169.00	CLP	2.84	1.72	0.00%	0.00%	15.97%	0.00%	0.00%	16.0%
Colombia	10 450.00	COP	7.61	3.27	0.00%	0.00%	15.97%	0.00%	0.00%	16.0%
Cuba	35.00	CUP	. . .	. . .	0.00%	0.00%	42.00%	0.00%	0.00%	42.0%
Dominica	27.54	XCD	16.11	10.20	0.00%	0.00%	13.04%	0.00%	3.35%	16.4%
Dominican Republic	72.94	DOP	3.30	1.44	0.00%	0.00%	15.25%	0.00%	0.00%	15.3%
Ecuador	2.09	USD	3.98	2.09	0.00%	0.00%	10.71%	0.00%	0.00%	10.7%
El Salvador	1.22	USD	2.65	1.22	0.00%	0.00%	11.50%	0.00%	0.00%	11.5%
Grenada	. . .	XCD	. . .	. . .	. . .	. . .	. . .	. . .	. . .	. . .
Guatemala	14.27	GTQ	3.61	1.86	0.00%	0.00%	10.71%	0.00%	0.00%	10.7%
Guyana	766.00	GYD	7.58	3.67	0.00%	0.00%	12.28%	0.00%	0.00%	12.3%
Honduras	29.60	HNL	2.80	1.21	0.00%	0.00%	13.04%	0.00%	0.00%	13.0%
Jamaica	644.40	JMD	9.18	5.15	0.00%	0.00%	17.70%	0.00%	0.57%	18.3%
Mexico	23.86	MXN	2.57	1.23	0.00%	0.00%	0.00%	0.00%	0.00%	0.0%
Panama	1.81	PAB	3.77	1.81	0.00%	5.00%	0.00%	0.00%	0.00%	5.0%
Paraguay	7 450.00	PYG	2.91	1.21	0.00%	0.00%	9.09%	0.00%	0.00%	9.1%
Peru	4.80	PEN	2.76	1.45	0.00%	16.95%	15.25%	0.00%	0.00%	32.2%
Saint Kitts and Nevis	5.95	XCD	3.01	2.20	0.00%	0.00%	0.00%	1.88%	2.26%	4.1%
Saint Lucia	. . .	XCD	. . .	. . .	. . .	. . .	. . .	. . .	. . .	. . .
Saint Vincent and the Grenadines	5.85	XCD	3.77	2.17	. . .	. . .	. . .	. . .	. . .	. . .
Suriname	13.89	SRD	5.04	1.86	0.00%	0.00%	0.00%	0.00%	0.00%	0.0%
Trinidad and Tobago	24.30	TTD	5.82	3.60	0.00%	0.00%	11.11%	0.61%	0.00%	11.7%
Uruguay	51.00	UYU	1.95	1.52	0.00%	0.00%	18.03%	0.00%	0.00%	18.0%
Venezuela (Bolivarian Republic of)	48 200.00	VES	71.62	. . .	0.00%	0.00%	13.79%	0.00%	0.00%	13.8%

Source: Prepared by the authors using the study data.

Notes:
. . .: No data available
ml: Milliliters
PPP: Purchasing power parity
Data only available for countries which completed PAHO SSB tax survey, i.e. all PAHO Member States in Latin America and the Caribbean except Argentina, the Bahamas, the Plurinational State of Bolivia, Costa Rica, Haiti, and Nicaragua.
[a] Brazil: Retail price and tax data representing only the State of Rio de Janeiro. However, all indirect taxes applied on sugar-sweetened beverages in Brazil are applied at federal level, except the value added tax which rate varies by State.
[b] Local currency codes: According to International Organization for Standardization, ISO 4217 currency names and code elements (https://www.iso.org/iso-4217-currency-codes.html).

Table S5. Retail price and detailed tax share estimates for the most sold brand of energy drink, 1000 ml, in Latin America and the Caribbean in 2019 (based on legislation in effect as of 31 March 2019)

Country	Retail price of an energy drink of the most sold brand, 1000 ml				Taxes as a percentage of retail price					
	Retail price in local currency	Local currency[b]	Retail price in international dollar (PPP)	Retail price in US dollar	Amount-specific excise	Ad valorem excise	Value-added/sales taxes	Import duties	Other taxes	Total tax
Antigua and Barbuda	4.06	XCD	1.97	1.50	0.00%	0.00%	13.04%	4.21%	8.27%	25.5%
Barbados	8.75	BBD	3.93	4.38	0.00%	0.85%	14.89%	1.42%	0.00%	17.2%
Belize	2.25	BZD	1.71	1.13	4.28%	0.00%	11.11%	14.05%	4.64%	34.1%
Brazil[a]	6.99	BRL	3.06	1.79	0.00%	2.30%	21.32%	0.00%	7.47%	31.1%
Chile	1 390.00	CLP	3.38	2.04	0.00%	7.03%	15.97%	0.00%	0.00%	23.0%
Colombia	1 381.58	COP	1.01	0.43	0.00%	0.00%	15.97%	0.00%	0.00%	16.0%
Cuba	. . .	CUP	. . .	. . .	. . .	. . .	. . .	. . .	. . .	. . .
Dominica	2.50	XCD	1.46	0.93	0.00%	0.75%	13.04%	0.00%	0.39%	14.2%
Dominican Republic	31.69	DOP	1.43	0.63	0.00%	0.00%	15.25%	0.00%	0.00%	15.3%
Ecuador	0.45	USD	0.86	0.45	0.00%	8.12%	10.71%	0.00%	4.42%	23.3%
El Salvador	0.33	USD	0.73	0.33	15.00%	6.68%	11.50%	0.00%	0.00%	33.2%
Grenada	. . .	XCD	. . .	. . .	. . .	. . .	. . .	. . .	. . .	. . .
Guatemala	3.21	GTQ	0.81	0.42	0.94%	0.00%	10.71%	0.54%	0.00%	12.2%
Guyana	193.00	GYD	1.91	0.93	0.00%	0.00%	12.28%	7.88%	5.18%	25.3%
Honduras	34.21	HNL	3.24	1.40	0.57%	0.00%	13.04%	0.00%	0.00%	13.6%
Jamaica	54.87	JMD	0.78	0.44	0.00%	0.00%	14.16%	0.00%	0.32%	14.5%
Mexico	10.29	MXN	1.11	0.53	2.84%	0.00%	13.79%	0.00%	0.00%	16.6%
Panama	1.48	PAB	3.08	1.48	0.00%	1.66%	0.00%	0.00%	0.00%	1.7%
Paraguay	12 000.00	PYG	4.70	1.94	0.00%	1.07%	9.09%	3.58%	0.00%	13.7%
Peru	1.66	PEN	0.95	0.50	0.00%	16.95%	15.25%	0.00%	0.00%	32.2%
Saint Kitts and Nevis	6.60	XCD	3.34	2.45	0.00%	2.24%	0.00%	7.12%	2.13%	11.5%
Saint Lucia	3.07	XCD	1.58	1.14	0.00%	0.00%	11.11%	0.00%	1.67%	12.8%
Saint Vincent and the Grenadines	2.86	XCD	1.84	1.06	0.00%	8.62%	13.79%	0.00%	17.50%	39.9%
Suriname	5.25	SRD	1.91	0.70	2.47%	0.00%	5.74%	8.89%	1.56%	18.7%
Trinidad and Tobago	7.79	TTD	1.87	1.15	0.00%	0.00%	11.11%	3.81%	0.00%	14.9%
Uruguay	75.00	UYU	2.87	2.24	1.83%	0.00%	18.03%	0.00%	0.00%	19.9%
Venezuela (Bolivarian Republic of)	22 000.00	VES	32.69	. . .	. . .	. . .	. . .	. . .	. . .	. . .

Source: Prepared by the authors using the study data.

Notes:
. . .: No data available
ml: Milliliters
PPP: Purchasing power parity
Data only available for countries which completed PAHO SSB tax survey, i.e. all PAHO Member States in Latin America and the Caribbean except Argentina, the Bahamas, the Plurinational State of Bolivia, Costa Rica, Haiti, and Nicaragua.
[a] Brazil: Retail price and tax data representing only the State of Rio de Janeiro. However, all indirect taxes applied on sugar-sweetened beverages in Brazil are applied at federal level, except the value added tax which rate varies by State.
[b] Local currency codes: According to International Organization for Standardization, ISO 4217 currency names and code elements (https://www.iso.org/iso-4217-currency-codes.html).

Table S6. Retail price and detailed tax share estimates for the most sold brand of water bottle, 500 ml, in Latin America and the Caribbean in 2019 (based on legislation in effect as of 31 March 2019)

Country	Retail price of a water bottle of the most sold brand, 500 ml				Taxes as a percentage of retail price					
	Retail price in local currency	Local currency[b]	Retail price in international dollar (PPP)	Retail price in US dollar	Amount-specific excise	Ad valorem excise	Value-added/sales taxes	Import duties	Other taxes	Total tax
Antigua and Barbuda	0.99	XCD	0.48	0.37	0.00%	0.00%	0.00%	0.00%	9.09%	9.1%
Barbados	1.95	BBD	0.88	0.98	0.00%	0.00%	14.89%	2.87%	0.00%	17.8%
Belize	1.00	BZD	0.76	0.50	19.25%	0.00%	11.11%	0.00%	0.00%	30.4%
Brazil[a]	0.81	BRL	0.36	0.21	0.00%	0.00%	40.06%	0.00%	0.00%	40.1%
Chile	218.44	CLP	0.53	0.32	0.00%	0.00%	15.97%	0.00%	0.00%	16.0%
Colombia	1 500.00	COP	1.09	0.47	0.00%	0.00%	15.97%	0.00%	0.00%	16.0%
Cuba	11.00	CUP	. . .	. . .	0.00%	0.00%	42.00%	0.00%	0.00%	42.0%
Dominica	1.92	XCD	1.12	0.71	0.00%	0.00%	13.04%	0.00%	0.00%	13.0%
Dominican Republic	7.61	DOP	0.34	0.15	0.00%	0.00%	0.00%	0.00%	0.00%	0.0%
Ecuador	0.22	USD	0.43	0.22	0.00%	0.00%	10.71%	0.00%	8.93%	19.6%
El Salvador	0.29	USD	0.64	0.29	0.00%	0.00%	11.50%	0.00%	0.00%	11.5%
Grenada	. . .	XCD	. . .	. . .	. . .	. . .	. . .	. . .	. . .	. . .
Guatemala	2.21	GTQ	0.56	0.29	1.81%	0.00%	10.71%	0.00%	0.00%	12.5%
Guyana	55.00	GYD	0.54	0.26	0.00%	0.00%	12.28%	0.00%	18.18%	30.5%
Honduras	8.00	HNL	0.76	0.33	0.00%	0.00%	13.04%	0.00%	0.00%	13.0%
Jamaica	44.62	JMD	0.64	0.36	0.00%	0.00%	14.16%	0.00%	0.32%	14.5%
Mexico	3.58	MXN	0.38	0.19	0.00%	0.00%	0.00%	0.00%	0.00%	0.0%
Panama	0.45	PAB	0.94	0.45	0.00%	0.00%	0.00%	0.00%	0.00%	0.0%
Paraguay	1 950.00	PYG	0.76	0.32	0.00%	0.00%	9.09%	0.00%	0.00%	9.1%
Peru	1.20	PEN	0.69	0.36	0.00%	0.00%	15.25%	0.00%	0.00%	15.3%
Saint Kitts and Nevis	1.13	XCD	0.57	0.42	0.00%	0.00%	0.00%	0.00%	0.00%	0.0%
Saint Lucia	0.81	XCD	0.42	0.30	0.00%	0.00%	11.11%	0.00%	0.00%	11.1%
Saint Vincent and the Grenadines	2.52	XCD	1.62	0.93	0.00%	0.00%	13.79%	0.00%	19.84%	33.6%
Suriname	2.82	SRD	1.02	0.38	9.21%	0.00%	0.00%	0.00%	0.00%	9.2%
Trinidad and Tobago	3.05	TTD	0.73	0.45	0.00%	0.00%	0.00%	0.00%	0.00%	0.0%
Uruguay	33.33	UYU	1.28	1.00	2.01%	0.00%	18.03%	0.00%	0.00%	20.0%
Venezuela (Bolivarian Republic of)	4 500.00	VES	6.69	. . .	0.00%	0.00%	13.79%	0.00%	0.00%	13.8%

Source: Prepared by the authors using the study data.

Notes:
. . .: No data available
ml: Milliliters
PPP: Purchasing power parity
Data only available for countries which completed PAHO SSB tax survey, i.e. all PAHO Member States in Latin America and the Caribbean except Argentina, the Bahamas, the Plurinational State of Bolivia, Costa Rica, Haiti, and Nicaragua.
[a] Brazil: Retail price and tax data representing only the State of Rio de Janeiro. However, all indirect taxes applied on sugar-sweetened beverages in Brazil are applied at federal level, except the value added tax which rate varies by State.
[b] Local currency codes: According to International Organization for Standardization, ISO 4217 currency names and code elements (http://www.iso.org/iso/home/standards/currency_codes.htm).

Figure S1. Descending order ranking of decomposed total tax share estimates for an internationally comparable brand of sugar-sweetened carbonated drink, small, 355ml, in Latin America and the Caribbean in 2019 (based on legislation in effect as of 31 March 2019)

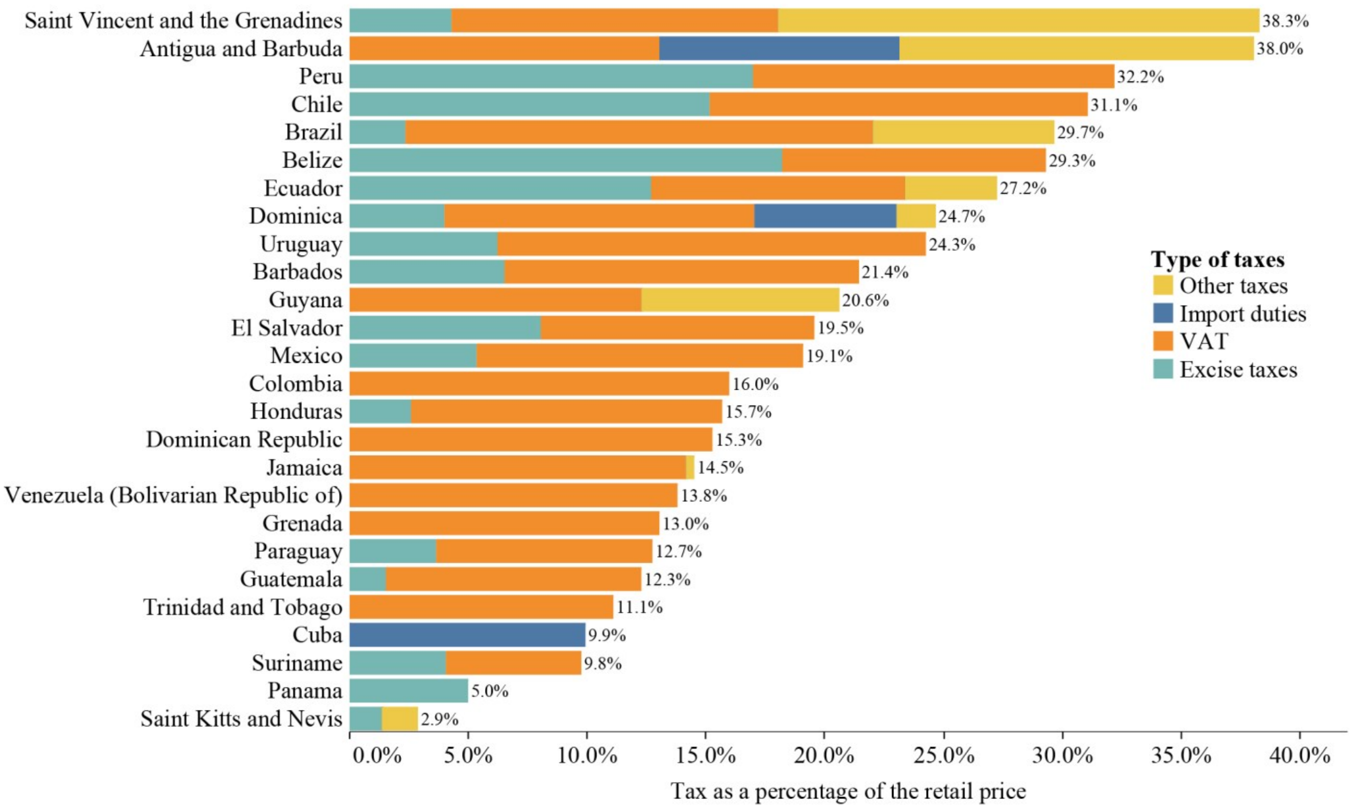

Source: Prepared by the authors using the study data
Notes:
ml: Milliliters
VAT: Value added/sales taxes
Data only available for countries which completed PAHO SSB tax survey, i.e. all PAHO Member States in Latin America and the Caribbean except Argentina, the Bahamas, the Plurinational State of Bolivia, Costa Rica, Haiti, and Nicaragua.
The internationally comparable brand selected for sugar-sweetened carbonates is regular Coca-Cola®.
Brazil: Retail price and tax data representing only the State of Rio de Janeiro. However, all indirect taxes applied on sugar-sweetened beverages in Brazil are applied at federal level, except the value added tax which rate varies by State.
Saint Lucia: No data available

Table S7. Sensitivity of excise and total tax share estimates for ad valorem excise taxes using producer price as tax base for locally produced sugar-sweetened beverages and the distribution margins mark-up assumption

	Distribution margins mark-up assumption											
	0%		10%		20%		30%		40%		50%	
	Excise tax share	Total tax share	Excise tax share	Total tax share	Excise tax share	Total tax share	Excise tax share	Total tax share	Excise tax share	Total tax share	Excise tax share	Total tax share
**Barbados**[a]												
Sugar-sweetened carbonated drink, small, 355 ml, internationally comparable brand	7.7%	22.6%	7.1%	22.0%	6.5%	21.4%	6.1%	21.0%	5.7%	20.6%	5.3%	20.2%
Sugar-sweetened carbonated drink, large, 1000 ml, internationally comparable brand	7.7%	22.6%	7.1%	22.0%	6.5%	21.4%	6.1%	21.0%	5.7%	20.6%	5.3%	20.2%
Fruit drink, 1000 ml, most sold brand	7.7%	22.6%	7.1%	22.0%	6.5%	21.4%	6.1%	21.0%	5.7%	20.6%	5.3%	20.2%
Sugar-sweetened milk drink, 1000 ml, most sold brand	7.7%	22.6%	7.1%	22.0%	6.5%	21.4%	6.1%	21.0%	5.7%	20.6%	5.3%	20.2%
**Brazil**[b]												
Sugar-sweetened carbonated drink, small, 355 ml, internationally comparable brand	2.7%	31.4%	2.5%	30.4%	2.3%	29.7%	2.2%	29.0%	2.0%	28.4%	1.9%	27.9%
Sugar-sweetened carbonated drink, large, 1000 ml, internationally comparable brand	2.8%	29.6%	2.6%	28.6%	2.4%	27.8%	2.2%	27.1%	2.1%	26.5%	2.0%	26.0%
Energy drink, 250 ml, most sold brand	2.7%	32.8%	2.5%	31.9%	2.3%	31.1%	2.1%	30.4%	2.0%	29.8%	1.9%	29.3%
**Dominica**[c]												
**Mexico**[d]												
**Paraguay**[e]												
Sugar-sweetened carbonated drink, small, 355 ml, internationally comparable brand	4.3%	13.4%	4.0%	13.0%	3.6%	12.7%	3.4%	12.5%	3.1%	12.2%	2.9%	12.0%
Sugar-sweetened carbonated drink, large, 1000 ml, internationally comparable brand	4.3%	13.4%	4.0%	13.0%	3.6%	12.7%	3.4%	12.5%	3.1%	12.2%	2.9%	12.0%
Fruit drink, 1000 ml, most sold brand	4.3%	13.4%	4.0%	13.0%	3.6%	12.7%	3.4%	12.5%	3.1%	12.2%	2.9%	12.0%

Source: Prepared by the authors using the study data.

Notes:
ml: Milliliters
The internationally comparable brand selected for sugar-sweetened carbonated drinks is regular Coca-Cola®.
This sensitivity analysis was not performed on sugar-sweetened milk drinks and bottled waters as none of the countries using the producer price as *ad valorem* excise tax base apply excise taxes on these beverages.
[a] Barbados: The most sold brand of energy drinks was imported, therefore the base for *ad valorem* excise tax used was the CIF value.

[b] Brazil: Retail price and tax data representing only the State of Rio de Janeiro. However, all indirect taxes applied on sugar-sweetened beverages in Brazil are applied at federal level, except the value added tax which rate varies by State. The excise tax does not apply on fruit drinks and sugar-sweetened milk drinks.
[c] Dominica: The country applies an *ad valorem* excise tax except for sugar-sweetened carbonated drinks, which are subject to an amount-specific excise tax. No excise taxes are applied on fruit drinks, sugar-sweetened milk drinks, and bottled waters. For energy drinks, the most sold brand was imported, therefore the base for *ad valorem* excise tax used was the CIF value.
[d] Mexico: The *ad valorem* component applies only to energy drinks. In 2019, it was applied only on energy drinks with more than 20 mg of caffeine per 100 ml. The most sold brand for which data was collected had a caffeine concentration below this threshold, therefore the *ad valorem* component did not apply. This caffeine concentration threshold was eliminated in 2020 by the law "Ley de Ingresos de la Federación para el Ejercicio Fiscal de 2020".
[e] Paraguay: The most sold brand of energy drinks was imported, therefore the base for *ad valorem* excise tax used was the CIF value.
