# Supplementary material for: Comparing taxes as a percentage of sugar-sweetened beverage prices in Latin America and the Caribbean
Source: Lancet Reg Health Am. 2022 Apr 21;11:100257. doi: 10.1016/j.lana.2022.100257 (PMC9290324; doi:10.1016/j.lana.2022.100257)
Supplement: Supplementary file 2 [file mmc2.docx]

***Editorial disclaimer:*** *This translation in Spanish was submitted by the authors and we reproduce it as supplied. It has not been peer reviewed. Our editorial processes have only been applied to the original abstract in English, which should serve as reference for this manuscript.*

**RESUMEN**

**Antecedentes**

Los impuestos selectivos se pueden utilizar para reducir el consumo de bebidas azucaradas (BAs), un factor de riesgo importante y evitable de enfermedades no transmisibles. Este estudio tuvo como objetivo comparar, a través de un indicador estandarizado pionero, el nivel de impuestos aplicados a las BAs como porcentaje del precio para diferentes categorías de bebidas en América Latina y el Caribe.

**Métodos**

Utilizamos un método desarrollado por la Organización Panamericana de la Salud, adaptado del método de monitoreo de impuestos al tabaco de la Organización Mundial de la Salud. El análisis se centró en la marca más vendida de cinco categorías de bebidas no alcohólicas. Los datos fueron recopilados a través de encuestas a los ministerios de finanzas y la revisión de las legislaciones tributarias vigentes a marzo de 2019.

**Resultados**

De los 27 países analizados, 17 aplican impuestos selectivos al consumo a las BAs. Entre estos países, las bebidas carbonatadas azucaradas grandes (6∙5%) tuvieron los niveles medianos de impuestos selectivos al consumo más altos como proporción del precio y las bebidas energéticas (2∙3%) la proporción más baja. En los países donde los impuestos selectivos se aplicaban a las aguas embotelladas, estos fueron más altos que a la mayoría de las BAs. En general, la proporción de los impuestos selectivos en los precios fue mayor en América Latina que en el Caribe. Incluidos todos los demás impuestos indirectos (ej., impuesto al valor agregado), las medianas de las participaciones fiscales totales se situaron entre el 12,8 % y el 17,5 % de los precios. Al menos dos países destinan parte de su recaudación por impuestos selectivos al consumo de BAs hacia fines de salud.

**Interpretación**

Los niveles de impuestos selectivos son generalmente bajos. Desde una perspectiva de salud pública, se podrían aumentar las tasas de impuestos y mejorar su diseño (ej., excluyendo las aguas embotelladas). Este método proporciona una forma factible e informativa de monitorear la tributación de BAs y podría replicarse en otras regiones y a lo largo del tiempo.

**Financiación**

Bloomberg Philanthropies a través de Global Health Advocacy Incubator.
